# Setmelanotide, a Novel, Selective Melanocortin Receptor-4 Agonist Exerts Anti-inflammatory Actions in Astrocytes and Promotes an Anti-inflammatory Macrophage Phenotype

**DOI:** 10.3389/fimmu.2019.02312

**Published:** 2019-10-04

**Authors:** Alwin Kamermans, Tom Verhoeven, Bert van het Hof, Jasper J. Koning, Lauri Borghuis, Maarten Witte, Jack van Horssen, Helga E. de Vries, Merel Rijnsburger

**Affiliations:** ^1^Department of Molecular Cell Biology and Immunology, MS Center Amsterdam, Amsterdam Neuroscience, Amsterdam UMC, Vrije Universiteit Amsterdam, Amsterdam, Netherlands; ^2^Department of Molecular Cell Biology and Immunology, Amsterdam UMC, Vrije Universiteit Amsterdam, Amsterdam, Netherlands

**Keywords:** multiple sclerosis, inflammation, melanocortin, melanocortin receptor-4, astrocyte, macrophage

## Abstract

To date, available treatment strategies for multiple sclerosis (MS) are ineffective in preventing or reversing progressive neurologic deterioration, creating a high, and unmet medical need. One potential way to fight MS may be by limiting the detrimental effects of reactive astrocytes, a key pathological hallmark for disease progression. One class of compounds that may exert beneficial effects via astrocytes are melanocortin receptor (MCR) agonists. Among the MCR, MC4R is most abundantly expressed in the CNS and several rodent studies have described that MC4R is—besides neurons—expressed by astrocytes. Activation of MC4R in astrocytes has shown to have potent anti-inflammatory as well as neuroprotective effects *in vitro*, suggesting that this could be a potential target to ameliorate ongoing inflammation, and neurodegeneration in MS. In this study, we set out to investigate human MC4R expression and analyze its downstream effects. We identified MC4R mRNA and protein to be expressed on astrocytes and observed increased astrocytic MC4R expression in active MS lesions. Furthermore, we show that the novel, highly selective MC4R agonist setmelanotide ameliorates the reactive phenotype in astrocytes *in vitro* and markedly induced interleukin−6 and −11 production, possibly through enhanced cAMP response element-binding protein (CREB) phosphorylation. Notably, stimulation of human macrophages with medium from astrocytes that were exposed to setmelanotide, skewed macrophages toward an anti-inflammatory phenotype. Taken together, these findings suggest that targeting MC4R on astrocytes might be a novel therapeutic strategy to halt inflammation-associated neurodegeneration in MS.

## Introduction

Multiple sclerosis (MS) is a progressive inflammatory and demyelinating disease of the central nervous system (CNS) and is one of the most common chronic neurological disease of young adults. To date, available treatment strategies are only partially effective in preventing and lack the potential to reverse progressive neurologic deterioration. In MS, evidence is increasing that astrocytes play a dominant role in the ongoing neuro-inflammation and neurodegeneration (1) and are able to aggravate inflammation as they express many factors such as complement components, cytokines, and chemokines to disrupt the blood-brain barrier, and attract immune cells into the CNS (2–6). Furthermore, reactive astrocytes are known to generate a glial scar at the lesion site, which inhibits remyelination, and axonal outgrowth (1, 7). On the other hand, astrocytes also secrete immunosuppressive molecules and exert neuroprotective properties, and the glial scar may form a physical barrier around areas of demyelination to prevent widespread tissue damage. Therefore, targeting reactive astrocytes may hold the key to counteract both ongoing neuro-inflammation as well as neurodegeneration in MS.

One class of compounds that may exert beneficial effects via astrocytes are melanocortin receptor agonists. Melanocortins, a group of highly-conserved neuropeptides that are cleaved in the pituitary gland from a common precursor, pro-opiomelanocortin (POMC), exert their action through activation of the family of G-protein coupled melanocortin receptors (MC1R-5R) (8). One of these melanocortins, alpha-melanocyte stimulating hormone (α-MSH), generally leads to weight loss due to a reduction in caloric intake and an increase in energy expenditure via MC4R activation (9, 10). Interestingly, α-MSH was also found to ameliorate disease in an animal model for MS, experimental autoimmune encephalomyelitis (EAE), by limiting inflammation in the CNS as well as in the periphery (11). Among the melanocortin receptors, MC4R is mostly expressed in the CNS including the thalamus, hypothalamus, cortex, hippocampus, and brainstem (12, 13). Besides its function in neurons, several studies have described that MC4R is functionally expressed by rodent astrocytes and, to a lesser extent, by the brains' resident macrophages, the microglia (14, 15).

Evidence is emerging that activation of MC4R in astrocytes may have potent anti-inflammatory as well as neuroprotective effects (14, 16, 17). Interestingly, selective MC4R agonists appear to be suited for the treatment of immune-mediated inflammatory diseases, without having the unfavorable side effects of more general melanocortin-related agents, such as corticosteroids (18). However, next to MC4R, α-MSH is also a full agonist for MC1R, and MC5R, which are both highly expressed in the periphery. Hence, treatment with α-MSH likely also leads to undesired peripheral side-effects. Together, this suggests that the astrocytic MC4R is a potential target to ameliorate ongoing inflammation and neurodegeneration in MS. However, to date, the expression of MC4R mRNA as well as protein in human astrocytes as well as their cellular distribution in MS brain tissue has not been studied in detail. Enhanced gene expression for the MC4R has been observed in active inflammatory MS lesions, however the cellular origin was not investigated (19). Additionally, *in vitro* evidence of the anti-inflammatory effects of MC4R activation are mainly built on LPS-induced inflammatory responses, while the inflammatory microenvironment seen in MS lesions is associated with increased cytokine levels including tumor necrosis factor alpha (TNF-α), and interferon gamma (IFN-γ) (20, 21).

In this study, we first show that MC4R mRNA is produced by astrocytes, using *in situ* hybridization chain reaction. Secondly, we identified increased astrocytic protein expression of the melanocortin receptor MC4R in active MS lesions. Furthermore, we showed that *in vitro* activation of the MC4R with setmelanotide ameliorated a reactive phenotype in astrocytes, and observed that astrocyte conditioned medium from setmelanotide stimulated astrocytes skewed macrophages toward an anti-inflammatory phenotype, which could limit ongoing damage, and eventually reduce clinical disability in MS (22, 23). Taken together, our novel findings suggest that targeting MC4R on astrocytes provide opportunities for the development of new treatments for MS.

## Materials and Methods

### Brain Tissue

Brain tissue from 17 donors with clinically diagnosed and neuropathological confirmed MS [*n* = 11, all secondary progressive MS (SPMS)] or non-demented controls (*n* = 6) was obtained at rapid autopsy and immediately frozen in liquid nitrogen. All parties received permission to perform autopsies, for the use of tissue and for access to medical records for research purposes from the UK MS Society Tissue Bank, Imperial College London and the Netherlands Brain Bank. All patients and controls, or their next of kin, had given informed consent for autopsy and use of their brain tissue for research purposes. Clinical data of patients and controls are listed in Table 1. Active lesions were immunohistochemically characterized as lesions with abundant immune cell infiltrates and extensive myelin loss.

**Table 1:** Patient details.

**Case**	**Age (years)**	**Gender**	**Post-mortem delay (h:min)**	**Lesion stage**
Ctrl 1	64	M	18:00	NA
Ctrl 2	35	M	22:00	NA
Ctrl 3	68	M	30:00	NA
Ctrl 4	77	M	22:00	NA
Ctrl 5	72	F	7:20	NA
Ctrl 6	59	M	8:00	NA
MS 1	35	F	09:00	A
MS 2	53	F	17:00	A
MS 3	39	F	09:00	A
MS 4	77	M	04:15	A
MS 5	47	M	10:00	A
MS 6	48	F	9:20	A, CA
MS 7	66	F	6:00	CA
MS 8	51	M	11:00	CA
MS 9	43	M	26:00	CA
MS 10	40	M	27:00	CA
MS 11	50	F	22:00	CA

*Clinical data of MS (all SPMS) patients and non-neurological controls. M, male; F, female; A, active lesions; CA, chronic active lesions; NA, non-applicable*.

### *In situ* Hybridization Chain Reaction

#### Human Brain Tissue

To reveal the cellular localization of MC4R mRNA in the human brain, *in situ* hybridization chain reaction (HCR) was performed on PFA-fixed brain slides from a control patient. Using *in situ* HCR, DNA probes complementary to mRNA targets carry DNA initiators that trigger chain reactions in which metastable fluorophore-labeled DNA hairpins self-assemble into tethered fluorescent amplification polymers. Sections (6 μm) were cut on a cryostat and mounted on superfrost plus glass slides (VWR international, Leuven, Belgium). Sections were stored at −80°C. RNA probes against MC4R and GAPDH (positive control) and buffers were obtained at Molecular Instruments (Los Angeles, CA, USA), and HCR was performed according the manufacturers protocol (24, 25). Sections were air-dried and epitope retrieval was done using citrate buffer pH 6.0. Then, slides were incubated with hybridization buffer for 10 min at 37°C followed by incubation with probe solution (2 pmol of probe diluted per 100 μL hybridization buffer) for 12–16 h in a humidified chamber at 37°C. Thereafter, slides were washed with wash buffer containing 25, 50, 75, and finally 100% SSCT (saline-sodium citrate 5X, diluted in H_2_O from 20X solution + 0.1% Tween-20). Amplification was performed by incubation with amplification buffer for 30 min at RT followed by incubation with fluorophore (647) RNA hairpins (6 pmol of h1 and 6 pmol of h2 diluted in 100 μL amplification buffer) for 12–16 h in a humidified chamber at RT. Subsequently, sections were stained with GFAP antibody (1:700, Sigma-Aldrich, Saint Louis, MO, USA) for 1 h and counterstained with DAPI (1:10,000). Images were made with a Leica DM6000 fluorescent microscope.

#### Cell Cultures

Human astrocytoma cells (U373) stably overexpressing MC4R+ were also labeled for MC4R mRNA and compared to empty vector controls (mock). Cells plated on coverslips were fixed with 4% PFA for 10 min. Thereafter they were permeabilized overnight at 4°C with 70% ethanol. Slides were then washed with 2xSSC and incubated with RNA probes against MC4R overnight at 37°C. The next they slides were washed with probe wash buffer and subjected to hairpins for 2 h. Images were made with a Leica DM6000 fluorescent microscope and mRNA amount was analyzed using ImageJ. Slides without probe but with fluorophore hairpins served as negative controls.

### Immunohistochemistry

Snap-frozen blocks of post-mortem normal control and MS brains were cut (6 μm) on a cryostat and mounted on superfrost plus glass slides (VWR international, Belgium). Sections were stored at −80°C. To determine MS lesion type, one section per case was stained for proteolipid protein (PLP, 1:500, Serotec, Kidlington, UK) and one for human leukocyte antigen–antigen D related (HLA-DR; MHCII, 1:2,000, in house antibody). Sections were defrosted at room temperature (RT), where after they were fixed in acetone for 10 min. After 3 washes with PBS, slides were incubated with antibodies for 1 h at RT. All antibodies were diluted in 1x phosphate-buffered saline (PBS) and 0.05% Tween-20. After 3 washes with PBS, slides were incubated with EnVision+ Dual Link System-HRP (Agilent DAKO, Santa Clara, CA, USA) for 30 min. 3,3′-diaminobenzidine (DAB) was used as a chromogen. Sections were counterstained with haematoxylin for 1 min and thoroughly washed with tap water for 3 min. Finally, sections were dehydrated in alcohol and xylene series and mounted with Entellan. Bright field images were taken with a Zeiss microscope (AXIO Scope A1, Carl Zeiss, Germany).

To reveal the cellular localization of MC4R, immunofluorescence triple-labeling was performed. Slides were fixed in acetone for 10 min and a blocking step was performed (30 min 10% normal goat serum). Thereafter, slides were incubated with the antibodies for MC4R (1:1,000, ab75506, Abcam, Cambridge, UK) and HLA-DR (MHCII) or biotinylated ULEX Europaeus Agglutinin1 (UEA-1) (1:1,000, Vector labs, Burlingame, CA, USA). Slides were washed and incubated with sheep-anti-rabbit alexa-488, sheep-anti-mouse-alex647 (1:400, Invitrogen, Carlsbad, CA USA), Cy3 labeled GFAP (1:700, Sigma-Aldrich), or streptavidin-647 (1:400, Invitrogen), respectively, diluted in 1x PBS/0.05% Tween-20 at RT for 1 h. After three washes with PBS, slides were incubated for 1 min with Hoechst (1:1,000) to visualize cellular nuclei and mounted with Mowiol. Z-stack images were taken with a 40x objective with a confocal microscope (Leica SP8 STED, Leica microsystems, Germany).

### Cell Treatments

Human astrocytoma cells (U373) were cultured in DMEM/F12 (ThermoFisher scientific, Waltham, MA, USA) containing 10% fetal calf serum (FCS, Life Technologies), and penicillin/streptomycin (50 mg/mL; ThermoFisher scientific) in 5% CO^2^ at 37°C.

U373 cells were incubated with human recombinant TNF-α and IFN-γ (both 4 ng/ml, Peprotech, London, UK). Setmelanotide (MedChemExpress, Monmouth Junction, NJ, USA) was used as a MC4R agonist and SHU9119 (Sigma-Aldrich) as a MC4R antagonist at concentrations indicated in the graphs. Cells were cultured in medium without FCS 4 h prior to the start of stimulations. Cells were treated with 0.001–10 μM setmelanotide for 1 h and thereafter with TNF-α and IFN-γ for 4 h. For blocking experiments, 10 μM SHU9119 (26) was added 30 min before addition of setmelanotide during 4 h.

### Retroviral-Induced Overexpression of MC4R

Human astrocytoma cells (U373) stably overexpressing MC4R (MC4R^+^ astrocytes) and empty vector control (Mock astrocytes) were generated by retroviral transductions. Expression vector encoding the human MC4R (pLenti-GIII-CMV-GFP-2A-Puro) was obtained from Applied Biological Materials (abm, Vancouver, Canada). HEK293FT cells were cultured in DMEM containing 10% FCS, 1% penicillin/streptomycin (50 mg/mL; ThermoFisher scientific) at 37°C in a 5% CO^2^ incubator. HEK293FT cells were transfected with calcium phosphate as a transfection reagent. Medium was refreshed 6 h post transfection. Supernatant containing virus was collected and virus was concentrated by centrifugation and stored at −80°C. For transduction of U373 cells, virus-containing supernatant was added dropwise to U373 cells in a 6 well-microplate. Virus supernatant was replaced with appropriate medium after 24 h incubation. Transduced cells were selected using puromycin (1:2,000). The effect of MC4R+ overexpression on cell viability and proliferation was assessed by CellTiter 96® Aqueous One Solution Cell Proliferation Assay (Promega, Madison, WI, USA) according to manufacturer's instructions.

### RNA Isolation and Real-Time Quantitative PCR

To assess gene expression of the MCR's in human MS lesions, normal appearing white matter (NAWM) (*n* = 5), active (*n* = 5), chronic active (*n* = 5), and white matter of controls (*n* = 5) were isolated from the brains described in Table 1. Lesions were outlined according to their PLP and LN3 status, using a sharp needle. Thereafter, 10 μm sections were cut, and lesion area and NAWM were collected separately in tubes and kept in liquid nitrogen. Messenger RNA isolation was conducted using the Qiagen RNeasy Lipid Tissue Mini Kit (Qiagen, Hilden, Germany) according to manufactures protocol.

Gene expression analysis in cell cultures was performed on subconfluent astrocytes in 24 well-microplates and messenger RNA was isolated using Trizol (Invitrogen) as described by the manufacturer. mRNA concentration and quality were measured using Nanodrop (Thermofisher scientific). cDNA synthesis was performed using the Reverse Transcription System kit (Promega) following manufacturers guidelines. RT-PCR was performed as described previously (27). Primers were all synthesized by Isogen life Sciences and sequences used are listed in Table 2. Obtained mRNA expression was normalized to the geometric mean of GAPDH, polrf2, and 18S in the human tissue samples and to GAPDH in human astrocytes.

**Table 2:** Primer sequences.

**Primer**	**Sequence**
MC1R	Forward 5′-TCTCCAGGGCTCACTAGCAT-3′ Reverse 5′-CTGCAGGAGTTTGCACATCG-3′
MC2R	Forward 5′-ATTCCTTCTCATTCATTTTGCCCA-3′ Reverse 5′-AAGTTAAAATCTCCCAATCACCTTC-3′
MC3R	Forward 5′-GCGACTACCTGACCTTCGAG-3′ Reverse 5′-TAGCGGAGCGCGTAAAAGAT-3′
MC4R	Forward 5′-AGC TCC TTG CTT GCA TCC AC-3′ Reverse 5′-TCC CAA CCC GCT TAA CTG TC-3′
MC5R	Forward 5′-CTT TGT GCG CCA CAT TGA CA-3′ Reverse 5′-GCC GTC ATG ATG TGG TGG TA-3′
CCL2	Forward 5′-AGT GTC CCA AAG AAG CTG TG-3′ Reverse 5′-AAT CCT GAA CCC ACT TCT GC-3′
CXCL10	Forward 5′-TTC AAG GAG TAC CTC TCT CTA G-3′ Reverse 5′-CTG GAT TCA GAC ATC TCT TCT C-3′
IL-6	Forward 5′-TGC AAT AAC CAC CCC TGA-3′ Reverse 5′-TGC GCA GAA TGA GAT GAG TTG-3′
IL-11	Forward 5′-GCG GAC AGG GAA GGG TTA AA-3′ Reverse 5′-GCG GCA AAC ACA GTT CAT GT-3′
GAPDH	Forward 5′-CCA TGT TCG TCA TGG GTG TG-3′ Reverse 5′-GGT GCT AAG CAG TTG GTG GTG-3′
Polr2f	Forward 5′-GAA CTC AAG GCC CGA AAG-3′ Reverse 5′-TGA TGA TGA GCT CGT CCA C-3′
18S	Forward 5′-TAC CAC ATC CAA GGA AGG CAG CA-3′ Reverse 5′-TGG AAT TAC CGC GGC TGC TGG CA-3′

### Western Blot

Cells were grown subconfluent in a 6 well-microplate and lysed using RIPA buffer [150 mM NaCl, 1% NP-40, 0.5% sodium doxycholate, 0.1% SDS, 25 mM Tris, 1× phosphostop, and 1× protease inhibitor (Roche, Almere, the Netherlands)]. Protein concentrations were measured with BCA assay according to manufacturer's protocol. A total of 10–15 μg protein was loaded onto SDS-PAGE gels (12.5%) along with a pre-stained protein marker (Precision plus, Bio-Rad Laboratories, Veenendaal, the Netherlands). Proteins were subsequently electrotransferred onto a nitrocellulose membrane (Bio-Rad, pore size 0.45 μm). The membrane was incubated with anti- CREB (1:500, #9104, Cell signaling) and anti-pCREB (1:300, #9198, cell signaling) and anti-α-tubulin as a loading control (1:400, S#SC1616, Santa Cruz, Dallas, TX, USA) in Odyssey blocking buffer (LI-COR, Lincoln, NE, USA) diluted 1:1 in TBS, after initial blocking with blocking buffer for 1 h at RT. Primary antibodies were detected by incubation with corresponding IRDye secondary antibodies (1:15,000) for 1 h at RT in blocking buffer and the Odyssey infrared imaging system (LI-COR). Intensity measurements of immunoreactivity were obtained using ImageJ software. Whole blots are shown in Supplementary Figure 3.

### ELISA

A commercial human SimpleStep enzyme-linked immunosorbent assay (ELISA) was used for detecting IL-11 and conducted according to manufacturer's protocol (ab189569, Abcam). For the detection of IL-6 levels, 96 wells-plates were coated overnight at 4°C with capture antibody (1 μg/mL), washed and blocked for 30 min with PBS-BSA (1%). Supernatant from astrocyte cultures or standard (100 μL/well) and secondary antibody (50 μL/well) were then added and incubated for 2 h at RT. After washing with PBST, streptavidin poly-HRP was added and incubated for 30 min at RT. Substrate was added to develop the staining (0.1 M NaAc/0.1 M citric acid pH4) for a maximum of 30 min. Then, 50 μL 0.8 M H_2_SO_4_ was added to stop the reaction. Optical density was measured with a plate reader at 450 nm. Samples for IL-11 were diluted 1:5 and for IL-6 1:3.

### Monocyte Isolation and Differentiation

Human blood monocytes were isolated from buffy coats of healthy donors (Sanquin Blood Bank, Amsterdam, The Netherlands) using Ficoll (Lymphoprep™, Axis-Shield, Oslo, Norway), and subsequent Percoll density gradient centrifugation. Monocytes were differentiated into macrophages in culture medium (IMDM, Gibco, ThermoFisher) containing 10% FCS, penicillin (100 IU/mL), streptomycin (50 mg/mL), and 50 ng/mL MCSF, for 6 days at 37°C/5% CO2. Macrophages were stimulated with astrocyte conditioned medium (ACM) from either mock or MC4R+ astrocytes for 3 days. To this aim, we stimulated astrocytes with setmelanotide and/or SHU9119 (10 μM) for 4 h, replaced the medium with macrophage culture medium and added the medium to the macrophages 24 h thereafter. This was repeated for 3 days. From the medium, 100 μL was collected in Eppendorf tubes and stored at −80°C for ELISA assays.

### Flow Cytometry

Lidocain (4 ng/mL) was added to macrophages and then cells were detached by gentle scraping, spun down, and collected in buffer (PBS plus 0.5% bovine serum albumin). Cells were incubated for 20 min at 4°C with appropriate antibodies, fluorescence minus one (FMO), and single stains were taken along for every antibody. Antibodies were as follows: CD14-AF700, CD68-PE-CY7 CD86-BV650, CD163-BV421, CD209-FITC, CD206-APC (Biolegend, San Diego, CA, USA). The cells were then washed twice with 100 μl of flow buffer, centrifuged, and resuspended in 200 μl flow buffer for analysis. Flow cytometry was performed with fluorescence-activated cell sorter (FACS) Calibur flow cytometer (BD Biosciences, San Jose, CA, USA). The cell populations were gated based upon FSC and SSC parameters and normalized to cells alone (without antibody) to adjust for cell-specific autofluorescence and a gate was set on the CD14 positive population (Supplementary Figure 4). Data were analyzed using FlowJo (FlowJo JCC, Ashland, OR, USA).

### Statistics

DAB images were color deconvoluted using Fuji to split the DAB and haematoxylin signals and the area fraction of the DAB staining (% immunopositive area of total area) was quantified using Image J version 1.48 (28). All data reflect mean ± SEM and all comparisons were statistically tested in GraphPad Prism 5.0 using either unpaired two-tailed Student's *t*-tests for comparing two experimental groups, or one-way analysis of variance (ANOVA) to compare more than two groups.

## Results

### Expression of Melanocortin Receptors in Human White Matter Samples and MS Lesions

**Figure 1:**
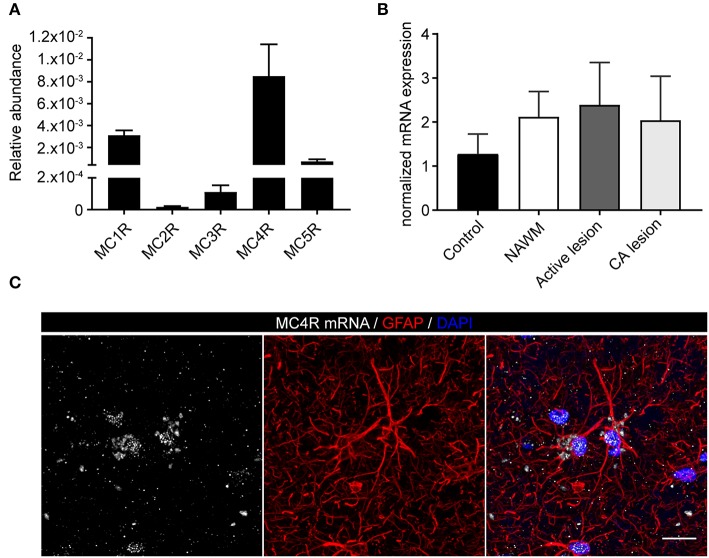
mRNA expression of melanocortin receptors in MS lesions. **(A)** Expression of MC1-5R mRNA in human white matter control samples shows abundant expression of MC4R (*n* = 5 per group). **(B)** No significant differences in mRNA expression of MC4R in control, normal appearing white matter (NAWM), active white matter lesions, and chronic active lesions as performed with one-way ANOVA. **(C)** *In situ* HCR reveals presence of MC4R mRNA (white) in GFAP-positive cells (red) in human brain tissue. Nuclei were stained in blue (DAPI). Scale-bar = 20 μm.

**Figure 2:**
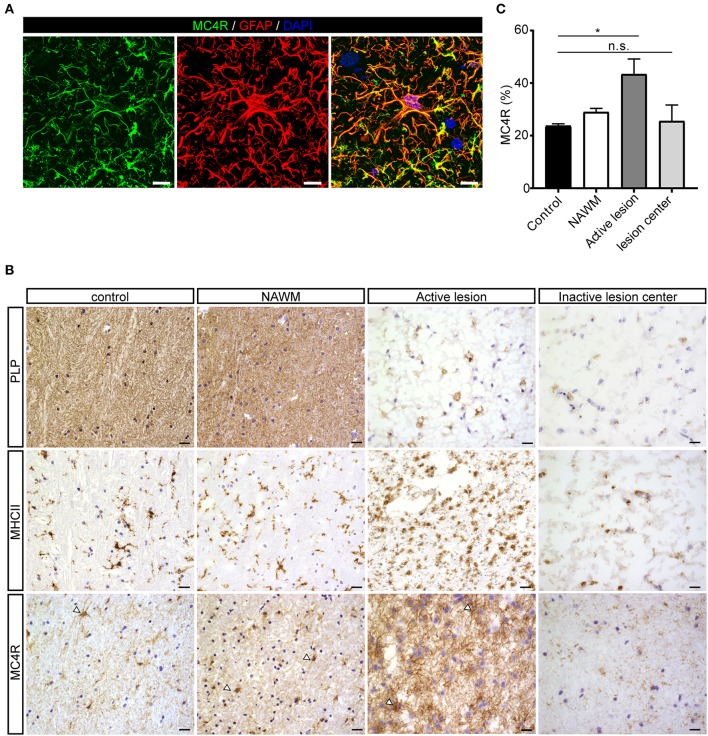
Protein expression of MC4R in MS lesions. **(A)** Double immunofluorescence labeling shows co-localization of MC4R (green) with GFAP-positive astrocytes (red). scale-bar = 5 μm. Nuclei were stained in blue (DAPI). **(B)** Immunostainings of representative control white matter, NAWM, an active lesion, and inactive lesion center. Active MS lesions are characterized by loss of proteolipid protein (PLP) and presence of MHCII positive leukocytes, while inactive lesions are characterized by a demyelinated core, with little presence of MHCII+ cells. MC4R is expressed in control and NAWM and increased immunoreactivity is observed in active lesions (open arrowheads, scale-bar = 25 μm). **(C)** Quantitative analysis (one-way ANOVA) of the immunoreactive area (% of total area) for MC4R in active lesions and chronic inactive lesion centers compared to NAWM and control (*n* = 3–5 per group). Data represent mean ± SEM. *p < 0.05.

To investigate the expression of melanocortin receptors, mRNA was isolated from control white matter tissue and well-characterized active white matter MS lesions. White matter lesions were identified and classified by the absence of myelin (proteolipid protein) and the presence of MHC class II^+^ cells as reported before (29). As expected, all MCR, except MC2R, were expressed in white matter lysates of control brain tissue, with MC4R being the most abundantly expressed MCR (Figure 1A). We next analyzed mRNA expression of MC4R in normal appearing white matter (NAWM), active and chronic active (CA) lesions. Compared to controls, we found a non-significant trend toward increased expression of MC4R in active lesions compared to control white matter, and NAWM (Figure 1B). To confirm the cellular source of MC4R, we performed *in situ* HCR to detect MC4R mRNA together with protein staining of glial fibrillary acidic protein (GFAP) as an astrocytic marker, and showed that MC4R mRNA is abundantly present in GFAP-positive astrocytes (Figure 1C). As expected, we also observed neuronal mRNA expression (Supplementary Figure 1A). GAPDH mRNA was used as positive control and present in all nuclei (Supplementary Figure 1B). We then used an antibody against MC4R and showed that astrocytes express MC4R protein (Figure 2A), and that MC4R is not expressed by MHCII positive cells and endothelial cells, as detected by UEA-1 (Supplementary Figures 1C,D). To further reveal the expression pattern of astrocytic MC4R protein in lesions, we performed immunohistochemical analyses and showed that immunoreactivity of MC4R was significantly increased in active lesions compared to NAWM and control white matter, while in inactive lesions similar expression of MC4R was observed as in NAWM, and control (Figures 2B,C).

### MC4R Activation Reduces Reactive Phenotype and Increases IL-6 and IL-11 Secretion in Astrocytes

U373 astrocytoma cells in which MC4R cDNA was stably transduced were used to further elucidate the role of MC4R in astrocyte function. Overexpression of MC4R resulted in a significant induction of MC4R levels at both mRNA (*in situ*; Supplementary Figures 2A,B, qPCR; Supplementary Figure 2C) and protein level (Supplementary Figure 2D). Overexpression of MC4R did not alter viability or proliferation as determined by MTS assay (Supplementary Figures 2E,F).

**Figure 3:**
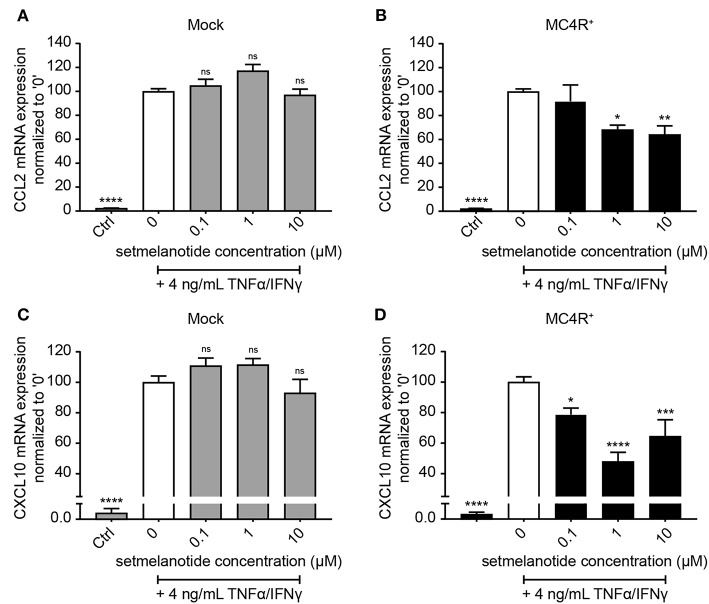
Setmelanotide decreases TNF-α/IFN-γ-induced chemokine expression in astrocytes. mRNA expression of CCL2 and CXCL10 are significantly increased upon treatment with TNF-α and IFN-γ, which was partially blocked by addition of setmelanotide in MC4R+ astrocytes at concentrations of 1 and 10 μM **(B,D)**, but not in mock astrocytes **(A,C)**. One-way ANOVA, *n* = 3 independent experiments with 3 technical replicates per experiment. *p < 0.05, ***p < 0.001, ****p < 0.0001 from control, compared to TNF-α and IFN-γ (“0”) conditions. **p < 0.01.

Astrocytes were subsequently exposed to TNF-α and IFN-γ, two pro-inflammatory cytokines described to be abundantly present during active demyelination in MS lesions, with or without the presence of setmelanotide, a selective MC4R agonist. We first analyzed the expression of two well-known reactive astrocyte markers C-C Motif Chemokine Ligand 2 (CCL2) and C-X-C motif chemokine 10 (CXCL10) (3, 30), which are involved in activation and recruitment of leukocytes into the CNS upon neuro-inflammation (31, 32). Treatment of mock and MC4R+ cells with TNF-α/IFN-γ resulted in a significant increase in mRNA expression of CCL2 and CXCL10 (Figures 3A–D). Importantly, treatment with setmelanotide strikingly reduced TNF-α/IFN-γ -induced expression of CCL2 and CXCL10 at doses of 1 and 10 μM in the MC4R+ astrocytes (Figures 3B,D), suggesting that the anti-inflammatory actions of setmelanotide are mediated via MC4R.

**Figure 4:**
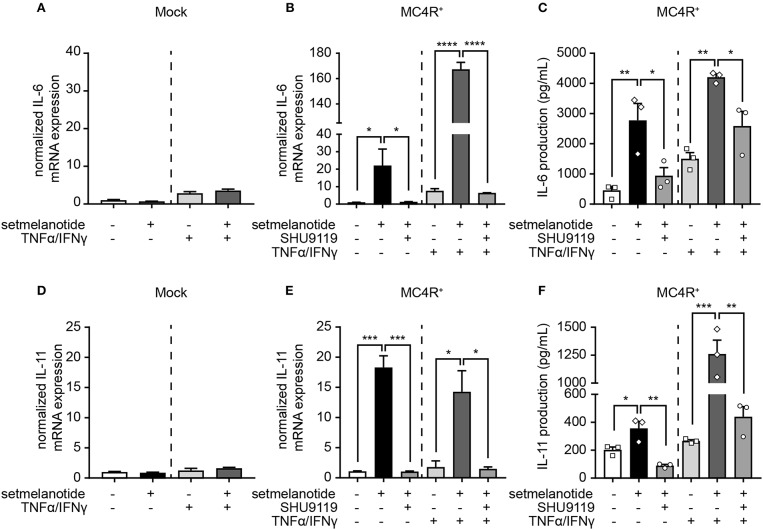
Setmelanotide increases IL-6 and IL-11 mRNA and protein secretion via MC4R. Setmelanotide (10 μM, black bars) significantly increased mRNA expression of IL-6 and IL-11 in MC4R+ astrocytes under control and inflammatory conditions **(B,E)** and not in mock cells **(A,D)**. Likewise, setmelanotide increased IL-6 and IL-11 protein production and secretion as measured in supernatant under basal and inflammatory conditions **(C,F)**. The effects of setmelanotide were completely blocked by MC4R antagonist SHU9119 (10 μM, gray bars, **B,C,E,F**). Student's *t*-test (mock) and One-way ANOVA (MC4R+), *n* = 2–3 independent experiments with 3 technical replicates per experiment for mRNA levels. For ELISA, supernatant was collected from 3 independent stimulations. *p < 0.05, **p < 0.01, ***p < 0.001, ****p < 0.0001.

IL-6 and IL-11 are produced by (activated) astrocytes in MS lesions and able to skew macrophages toward an anti-inflammatory (M2) phenotype (33). We therefore stimulated mock and MC4R+ astrocytes with setmelanotide and investigated the gene expression of IL-6 and IL-11, both under basal, and inflammatory conditions. Setmelanotide treatment induced a significant increase in IL-6 and IL-11 mRNA levels in MC4R+ astrocytes (Figures 4B,E), but had no effect on mock cells (Figures 4A,D). The increase in IL-6 and IL-11 was prevented by pretreatment with SHU9119, an MC4R antagonist (Figures 4B,E). Effects were observed both in unstimulated as well as TNF-α/IFN-γ stimulated astrocytes. Importantly, secretion of IL-6, and IL-11 was also increased upon setmelanotide treatment in MC4R+ cells (Figures 4C,F). Three other members of the IL-6 family [leukemia inhibitory factor (LIF), oncostatin M (OSM), ciliary neurotrophic factor (CNTF)] did not show an increase in mRNA levels after setmelanotide exposure (data not shown).

### Setmelanotide Induces CREB Phosphorylation in Astrocytes

**Figure 5:**
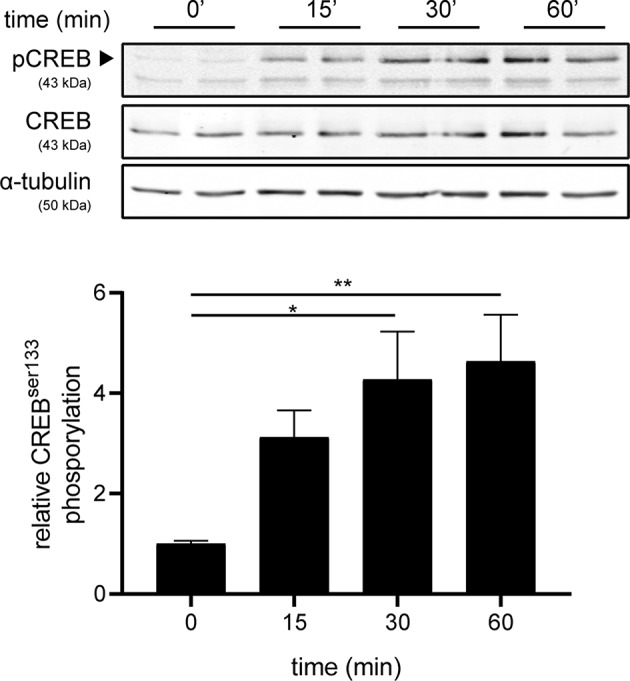
Setmelanotide time-dependently induces CREB phosphorylation. Representative western blots of MC4R+ astrocytes treated with setmelanotide (10 μM), lysed at 15, 30, and 60 min after stimulation. α-Tubulin was used as a protein loading control for both pCREB and CREB. Quantification of CREB phosphorylation relative to total CREB revealed significantly increased phosphorylation of CREB after setmelanotide stimulation. *n* = 3 independent experiments. One-way ANOVA. *p < 0.05, **p < 0.01.

Next, we investigated the mechanism underlying increased production of IL-6 and IL-11 upon setmelanotide exposure. To date, the main signaling pathway of MC4R involves activation of G protein αs subunit (Gαs) and subsequently phosphorylation of cAMP response element-binding protein (CREB) (34) can result in production of IL-6 and IL-11 (35). MC4R+ astrocytes were treated with 10 μM setmelanotide and CREB phosphorylation (pCREB) was analyzed by western blot after 15, 30, and 60 min of stimulation. Setmelanotide significantly induced pCREB in time without altering total CREB levels (Figure 5).

### Conditioned Medium of Setmelanotide Treated Astrocytes Skews Human Macrophages Toward an Anti-Inflammatory Phenotype

**Figure 6:**
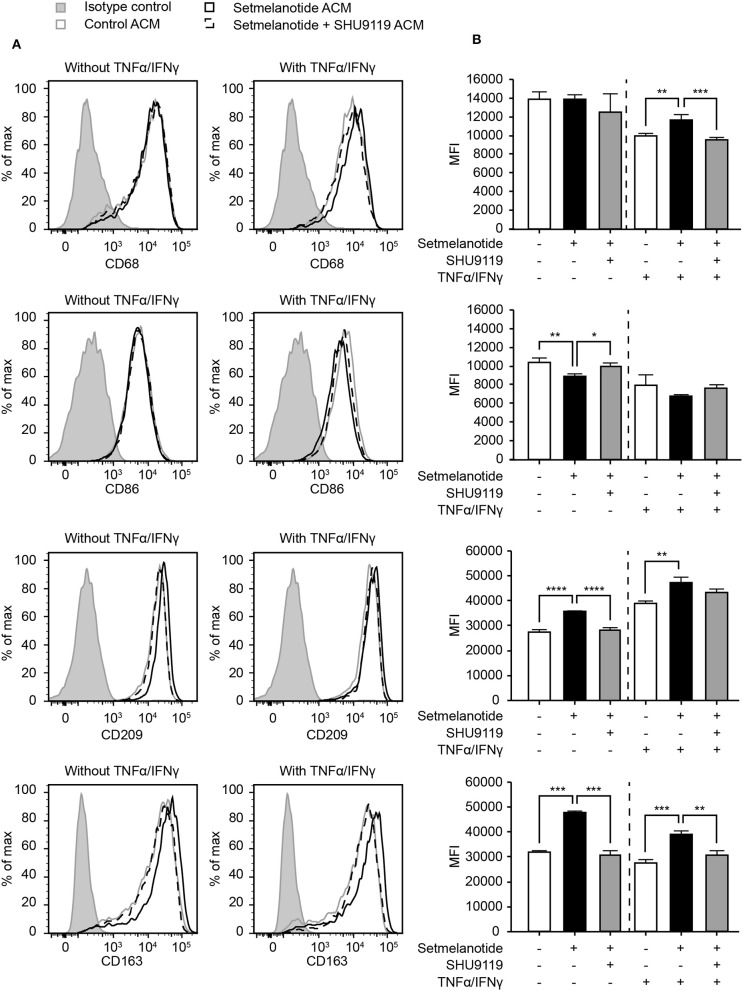
Astrocyte condition medium from setmelanotide treated astrocytes alters phenotype of human macrophages. **(A)** Macrophages were cultured in the presence of astrocyte condition medium from astrocytes and the expression of markers was analyzed by flow cytometry (*n* = 3). Gray line represents macrophages cultured in the presence of ACM from non-treated astrocytes, black line represents macrophages cultured in the presence of ACM from setmelanotide treated astrocytes. Dotted line represents macrophages cultured in the presence of ACM from astrocytes treated with both setmelanotide and SHU9119. Left histograms indicate ACM from astrocytes cultured without TNF-α/IFN-γ, right histograms with TNF-α/IFN-γ. **(B)** Quantification of mean fluorescent intensity (MFI) of CD86, CD209, CD163, and CD206. Human macrophages treated with ACM from setmelanotide treated astrocytes reduced CD86, increased CD209, and CD163, and decreased CD206 surface expression. Macrophages treated with ACM from setmelanotide treated reactive astrocytes (+TNF-α/IFN-γ) showed increased CD68, CD209, and CD163 expression and a decrease in CD206. Effects of setmelanotide were blocked upon pretreatment with MC4R antagonist SHU9119. One-way ANOVA, *p < 0.05, **p < 0.01, ***p < 0.001, ****p < 0.0001.

Reactive astrocytes are known to produce many factors such as cytokines, chemokines, and complement components, that influence inflammation, and can alter the course of MS (2). Both IL-6 and IL-11 have been shown to be present in MS lesions, released by astrocytes, and are involved in skewing macrophages toward an anti-inflammatory (M2) phenotype (33, 36, 37). We hypothesized that stimulation of astrocytes with setmelanotide, which results in increased IL-6 and IL-11 secretion, could affect the macrophage phenotype. Addition of conditioned medium from setmelanotide-treated astrocytes to macrophages resulted in a significant decrease in pro-inflammatory marker such as CD86 (M1 marker) and an increase in CD163 and CD209 (M2) protein expression, both under basal conditions as well as after stimulation with TNF-α/IFN-γ. This indicates that setmelanotide treated astrocytes secrete factors that are able to modulate macrophage polarization into an anti-inflammatory phenotype (Figures 6A,B). CD68 expression was not different in the conditions without cytokine stimulation but was however increased when stimulated with setmelanotide and cytokines.

## Discussion

In the present study, we are the first to show the presence of melanocortin receptor 4 (MC4R) mRNA, as well as protein in human astrocytes, and showed increased astrocytic MC4R immunoreactivity in human active MS lesions. Increased MC4R mRNA expression has been described in active inflammatory MS lesions, however the cellular origin yet remained unknown (19). We furthermore showed that MC4R activation with setmelanotide, a novel, selective MC4R agonist, reduced inflammation-driven chemokine expression and markedly induced the production of the interleukins IL-6 and IL-11 under unstimulated and inflammatory conditions, presumably by increasing CREB phosphorylation. Stimulation of human macrophages with astrocyte medium of setmelanotide treated astrocytes, skewed macrophages toward an anti-inflammatory phenotype, which could limit a local inflammatory response in MS. Taken together, we are the first to show that targeting astrocytic MC4R limits neuroinflammation by inhibition of inflammatory genes, activation of the cytoprotective IL-6 cytokine family and by modulation of macrophage phenotype,.

Tissue lysates provide valuable information about overall expression of genes and differences thereof between normal and affected tissue, but are composed of a mix of different cell types of which the immune cell component in active lesions dominates the protein content of the lysate. We therefore further looked at which cells in the tissue expressed MC4R protein and found that astrocytes highly expressed MC4R mRNA and protein, which was increased in active lesions.

It has been shown that the endogenous MCR ligand α-MSH, inhibits lipopolysaccharide (LPS)-induced TNF-α production in astrocytes, which was proposed to be mediated via MC1R (38). In addition, α-MSH (analogs) reduced LPS/IFN-γ-induced nitric oxide production and the expression of inducible nitric oxide synthase (iNOS) in cultured primary astrocytes, which was blocked by an MC4R antagonist (14, 17). This illustrates the potency of MC4R activation in reducing an inflammatory response. However, LPS stimulation does not represent a relevant stimulus to mimic inflammatory processes as seen in MS. Various studies have shown increased (local) production of cytokines including TNF-α and IFN-γ in inflammatory MS lesions (20, 21). We show that activation of MC4R with setmelanotide robustly decreased TNF-α/IFN-γ-mediated induction of astrocytic CXCL10 and CCL2 and increased IL-6 and IL-11 secretion under basal and inflammatory conditions. CXCL10 and CCL2 play a role in attracting T cells and monocytes, respectively, and have been strongly implicated in disease in experimental autoimmune encephalomyelitis (EAE), an animal for MS. Deleting these chemokines in astrocytes have proven beneficial in reducing immune cell infiltration in the CNS and improving or delaying clinical symptoms (39, 40).

Infiltrating macrophages as well as microglia have been identified as major effectors of demyelination in both MS and animal models for MS (41). In animal models, pro-inflammatory M1 macrophages worsen neurological symptoms, whereas M2 macrophages promote remyelination (22), and *ex vivo* administration of M2 macrophages has been shown to reduce clinical disability (23). In human macrophages, IL-6 induces an anti-inflammatory cytokine profile, characterized by enhanced IL-4 and IL-10 production and decreased IL-1β secretion (42). It furthermore skews human monocytes into an anti-inflammatory M2 phenotype characterized by high CD163 and low CD86 surface expression (43). IL-11 reduces release of TNF-α, IL-1β, IL-12, and nitric oxide by macrophages and also induces M2 differentiation of macrophages/microglia after intranasal administration in mice [for review see (33)]. These results indicate that IL-6 and IL-11 promote an anti-inflammatory M2 phenotype in macrophages/microglia, which could limit a local inflammatory response in MS. Reactive astrocytes produce IL-6 in both active and chronic active MS plaques, which correlated negatively with oligodendrocyte loss, suggesting a protective role of this cytokine in myelin repair (36). IL-11 has been shown to be increased in astrocytes in inactive lesion borders and potentiates oligodendrocyte survival and maturation, and myelin formation *in vitro* (37), and reduced demyelination in EAE mice (44). Thus, astrocytic stimulation of IL-6 and IL-11 with setmelanotide could prove beneficial in targeting both (infiltrated) macrophages as well-neurodegenerative processes.

Reactive astrocytes highly express many complement components, and release cytokines, and chemokines that signal to microglia and various other immune cell types including monocyte derived macrophages, to attract them into the CNS or regulate their immune functions, and can thereby influence the course of disease (2). Together with the increased IL-6 and IL-11 production in astrocytes we show that medium of astrocytes stimulated with setmelanotide skews macrophages toward a less inflammatory phenotype, as determined by upregulation of CD163 and CD209, two anti-inflammatory M2 markers, and downregulated CD86, a M1 macrophage marker. Macrophages in MS have been shown to obtain an intermediate phenotype with high expression of CD68, CD86, and CD163 (45), skewing macrophages locally toward a protective M2 phenotype has been shown to promote remyelination (22).

MC4R is a G-protein coupled receptor (GPCR), which signals via a cAMP dependent pathway to promote CREB phosphorylation (16). Besides CREB, cAMP can also activate other transcription factors, including NF-κB, activator protein (AP)-1, and CCAAT/enhancer binding protein (C/EBP) (46). CCL2 and CXCL10 production are induced by activation of NF-κB in astrocytes (47, 48). Interestingly, it was shown that MC4R agonists inhibit nuclear translocation of NF-κB, thereby preventing NF-κB activation (49). It is thus likely that the reduced reactive profile of TNF-α/IFN-γ treated astrocytes upon setmelanotide stimulation is partly due to reduced activation of NF-κB. Besides CXCL10 and CCL2, IL-6, and IL-11 are also under control of NF-κB (50, 51). However, since we observed an increased expression and secretion of IL-6 and IL-11 makes it unlikely that NF-κB is involved. Based on the induced phosphorylation CREB upon the activation of MC4R with setmelanotide, we postulate that this pathway is more dominant in the regulation of secretion of IL-6 and IL-11.

α-MSH and the stable analog NDP-MSH have both proven to be effective in ameliorating clinical symptoms in an experimental animal model of MS by limiting inflammation and neurodegeneration, which was attributed to MC1R signaling (11). α-MSH is, next to MC4R, a full agonist of MC1R and MC5R, which are both highly expressed in the periphery. Hence treatment with α-MSH likely also has peripheral effects. Selective MC4R agonists have proven to be excellent candidates in the treatment of immune-mediated inflammatory diseases, without having the side effects of corticosteroids (18). MC4R is the most widespread melanocortin receptor in the CNS including astrocytes. Therefore, a more selective MC4R agonist like setmelanotide could have beneficial effects with limited side-effects. Future studies are warranted to study the therapeutic efficacy of setmelanotide in experimental animal models of MS and whether astrocyte-specific MC4R knockout is able to block these effects. Setmelanotide has shown to be well-tolerated in a phase 2 clinical trial in obese individuals with a rare genetic disorder, and mouse knock-out studies have shown that setmelanotide-associated weight loss is regulated via MC4R, which is mainly attributed to MC4R neurons of the hypothalamus (52, 53). Our findings could set the stage for setmelanotide as a new therapeutic for inflammation associated neurodegeneration in MS.

## Data Availability Statement

All datasets generated for this study are included in the manuscript/Supplementary Files.

## Ethics Statement

The studies involving human participants were reviewed and approved by Dutch brain bank. The patients/participants provided their written informed consent to participate in this study.

## Author Contributions

MR, AK, HV, and JH designed and interpreted the experiments. MR, AK, TV, LB, and BH performed the experiments. JK set up the *in situ* hybridization protocol and helped with the *in situ* experiments. MR and AK analyzed the data and wrote the paper. MW collected the human brain samples and clinical data. HV, JH, and MW provided intellectual inputs and critically reviewed the manuscript. All authors have read and approved the manuscript.

### Conflict of Interest

The authors declare that the research was conducted in the absence of any commercial or financial relationships that could be construed as a potential conflict of interest.
