# Supplementary material for: Setmelanotide, a Novel, Selective Melanocortin Receptor-4 Agonist Exerts Anti-inflammatory Actions in Astrocytes and Promotes an Anti-inflammatory Macrophage Phenotype
Source: Front Immunol. 2019 Oct 4;10:2312. doi: 10.3389/fimmu.2019.02312 (PMC6788433; doi:10.3389/fimmu.2019.02312)
Supplement: Supplementary file 1 [file Presentation_1.pdf]

Supplementary material (total: 4 figures)

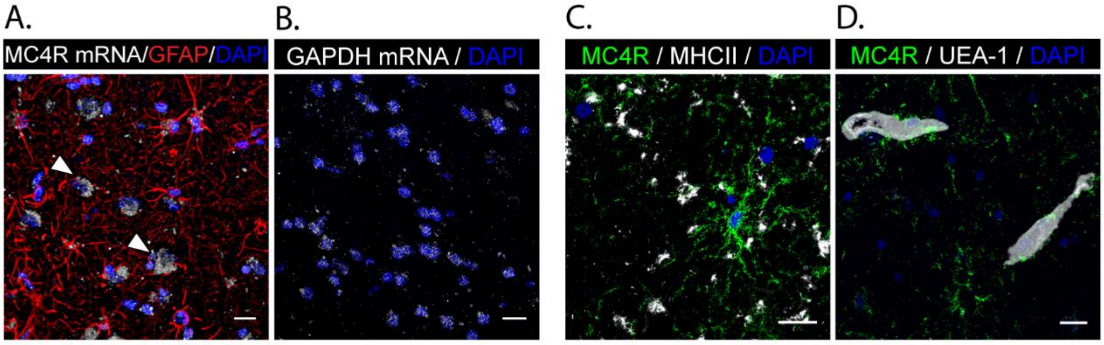

Supplementary figure 1. MC4R mRNA (white) is expressed on GFAP positive astrocytes (red) and on neurons (white arrows)(**A**). Positive control (GAPDH, white) for *in situ* HCR in human brain sections (**B**). Double immunofluorescence labelling shows no co-localization of MC4R protein (green) with MHCII positive cells (white, **C**) nor endothelial cells (UEA-1, white, **D**). Nuclei are stained blue. Pictures are taken in white matter control tissue. Scale-bar = 25 µm.

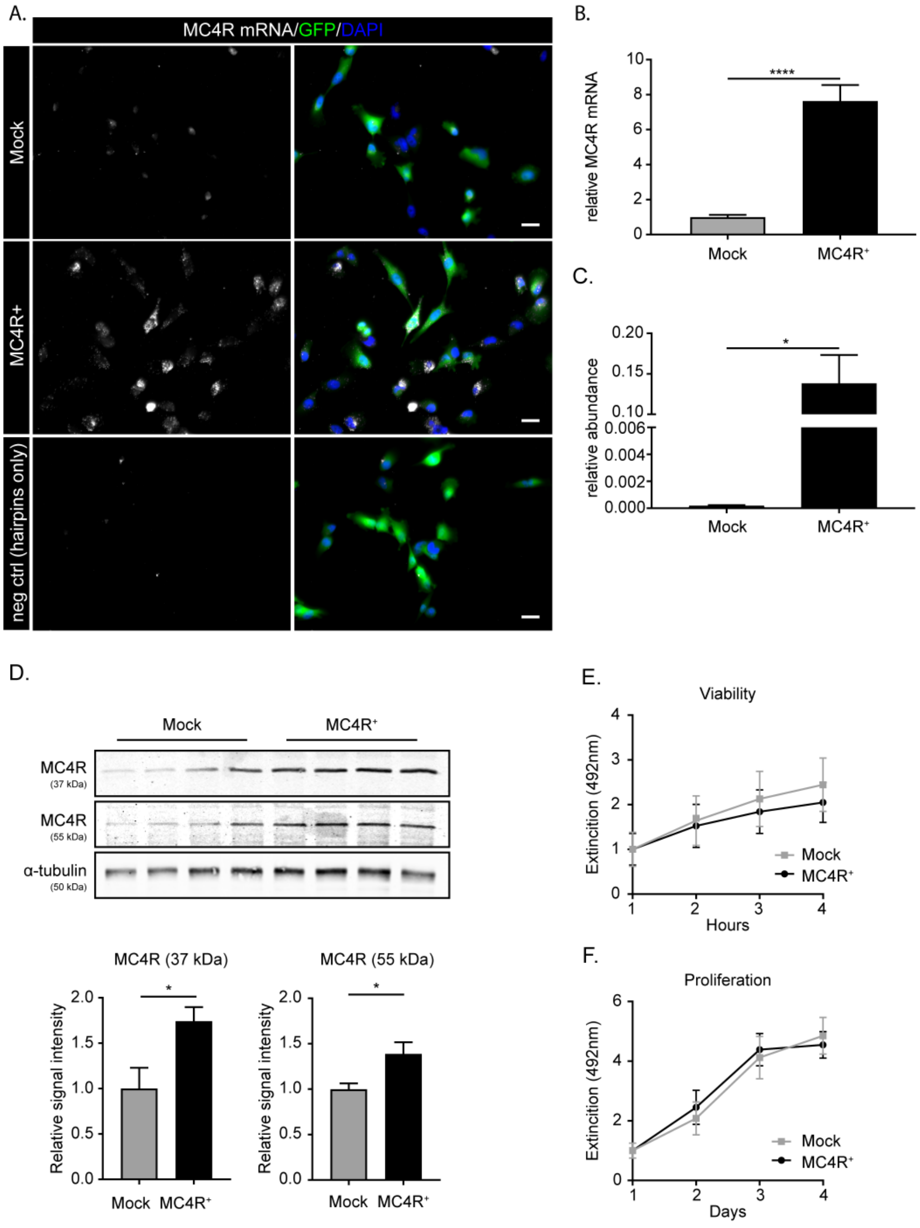

Supplementary figure 2. (**A**) representative pictures of MC4R mRNA in mock and MC4R+ u373 cells after *in situ* HCR. Negative controls, cells that were incubated only with hairpins, showed no signal. Scale-bar = 25 µm. (**B**) quantification of MC4R mRNA HCR corrected for DAPI signal. At least 60 cells were quantified per group. (**C**) Expression of MC4R relative to GAPDH with qPCR in mock and MC4R+ U373 cells shows increased expression of MC4R in MC4R+ U373 astrocytes (3 biological replicates with n = 3 per group). (**D**) MC4R protein expression in mock and MC4R+ cells. Quantification showed that MC4R expression is significantly increased in MC4R+ U373 cells compared to mock U373 cells. As described in the datasheet, the anti-MC4R antibody detects the unglycosylated protein at 37kDa and a glycosylated protein at 55kDa. 2 biological replicates with n = 4 per group. (**E**) Manipulation of MC4R expression does not affect cell viability and proliferation of U373 astrocytoma cells (n = 3). Viability was assessed over 4 hrs. (**F**) proliferation was assessed over 4 days. Data represent normalized mean extinction at 492nm. Data were analyzed using students' t-test. *$p < 0.05$, ****$p < 0.0001$.

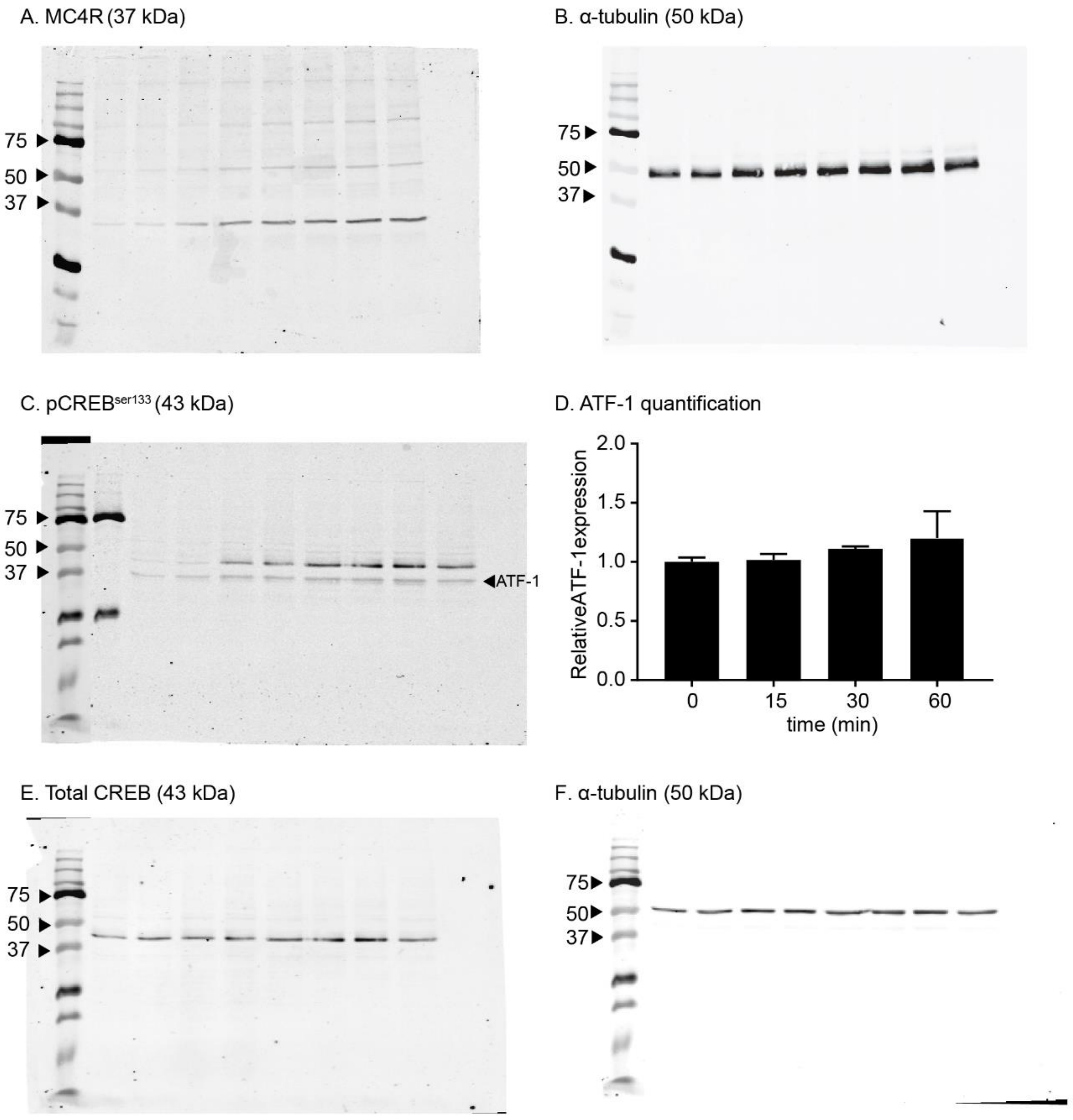

Supplementary figure 3. Representative blots for MC4R (**A**) and α-tubulin (**B**). Blot for pCREB blot shown in (**C**), an extra band was observed, antibody also detects the phosphorylated form of the CREB-related protein, Cyclic AMP-dependent transcription factor (ATF-1), quantified in (**D**) using one-way ANOVA. Blots for total CREB (**E**) and α-tubulin (**F**). All antibodies showed bands at their expected size.

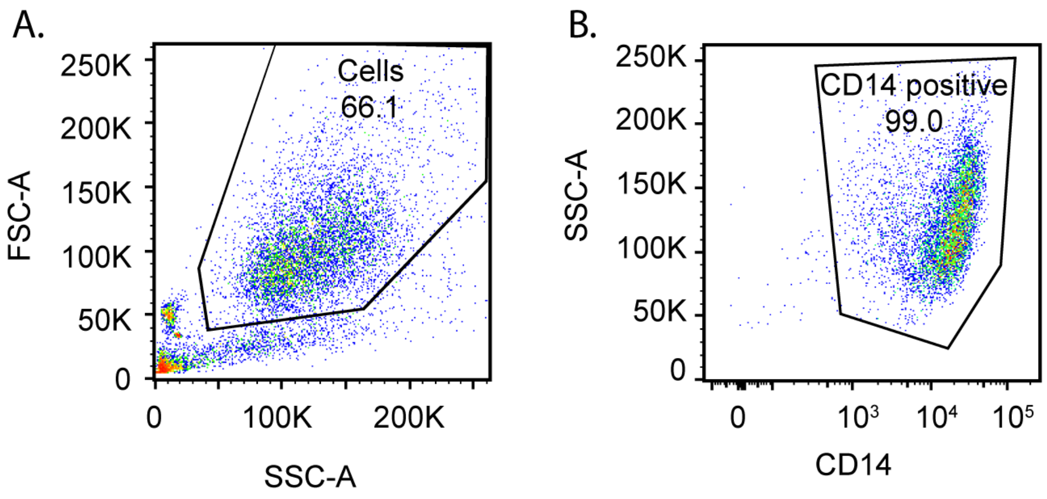

Supplementary figure 4. Gating strategy for FACS analysis in human macrophages. Forward (FSC) and side scatter (SSC) density plots were used to exclude debris (**A**), then cells were gated on CD14 positive (macrophages) cells (**B**).
